# Nup153 and Nup98 bind the HIV-1 core and contribute to the early steps of HIV-1 replication

**DOI:** 10.1186/1742-4690-10-S1-P111

**Published:** 2013-10-11

**Authors:** Francesca Di Nunzio, Thomas Fricke, Annarita Miccio, Jose Carlos Valle-Casuso, Patricio Perez, Philippe Souque, Ermanno Rizzi, Marco Severgnini, Fulvio Mavilio, Pierre Charneau, Felipe Diaz-Griffero

**Affiliations:** 1 Department of Virology, Institut Pasteur, Paris, France; 2 Department of Microbiology and Immunology, Albert Einstein College of Medicine, Bronx, NY, USA; 3 University of Modena e Reggio Emilia, Centro di Medicina Rigenerativa, Modena, Italy; 4 Institute of Biomedical Technologies, CNR, Milano, Italy

## 

The early steps of HIV-1 replication involve the entry of HIV-1 into the nucleus, which is characterized by viral interactions with nuclear pore components. HIV-1 developed an evolutionary strategy to usurp the nuclear pore machinery and chromatin in order to integrate and efficiently express viral genes. We investigated the role of nucleoporins 153 and 98 (Nup153 and Nup98) in infection of human Jurkat lymphocytes by HIV-1. We showed that Nup153-depleted cells exhibited a defect in nuclear import, while depletion of Nup98 caused a slight defect in HIV integration. To explore the biochemical viral determinants for the requirement of Nup153 and Nup98 during HIV-1 infection, we tested the ability of these nucleoporins to interact with HIV-1 cores. Our findings showed that both nucleoporins bind HIV-1 cores suggesting that this interaction is important for HIV-1 nuclear import and/or integration. Distribution analysis of integration sites in Nup153-depleted cells revealed a reduced tendency of HIV-1 to integrate in intragenic sites. Nup153 depletion reduces HIV-1 integration preferences for chromosomal regions rich in genes and associated features such as CpG islands, DNAase I hypersensitive sites, thus suggesting that Nup153 influences HIV-1 integration in transcriptional units and in regions characterized by an open chromatin configuration. The surrounded chromatin to integrated provirus in part could account for the large infectivity defect observed in Nup153-depleted cells. Overall our work suggests that the defect on infectivity observed for Nup153-depleted cells is due to the contribution of Nup153 to nuclear import and integration, suggesting a link between these two steps.

